# Human coronavirus OC43 3CL protease and the potential of ML188 as a broad-spectrum lead compound: Homology modelling and molecular dynamic studies

**DOI:** 10.1186/s12900-015-0035-3

**Published:** 2015-04-28

**Authors:** Michael Berry, Burtram Fielding, Junaid Gamieldien

**Affiliations:** South African National Bioinformatics Institute/ MRC Unit for Bioinformatics Capacity Development, University of the Western Cape, Bellville, South Africa; Molecular Biology and Virology Laboratory, Department of Medical Biosciences, University of the Western Cape, Bellville, South Africa

**Keywords:** Human coronavirus, OC43, 3CL^pro^, Homology modelling, Molecular dynamics

## Abstract

**Background:**

The coronavirus 3 chymotrypsin-like protease (3CL^pro^) is a validated target in the design of potential anticoronavirus inhibitors. The high degree of homology within the protease’s active site and substrate conservation supports the identification of broad spectrum lead compounds. A previous study identified the compound ML188, also termed 16R, as an inhibitor of the Severe Acute Respiratory Syndrome coronavirus (SARS-CoV) 3CL^pro^. This study will detail the generation of a homology model of the 3CL^pro^ of the human coronavirus OC43 and determine the potential of 16R to form a broad-spectrum lead compound. MODELLER was used to generate a suitable three-dimensional model of the OC43 3CL^pro^ and the Prime module of Schrӧdinger predicted the binding conformation and free energy of binding of 16R within the 3CL^pro^ active site. Molecular dynamics further confirmed ligand stability and hydrogen bonding networks.

**Results:**

A high quality homology model of the OC43 3CL^pro^ was successfully generated in an active conformation. Further studies reproduced the binding pose of 16R within the active site of the generated model, where its free energy of binding was shown to equal that of the 3CL^pro^ of SARS-CoV, a receptor it is experimentally proven to inhibit. The stability of the ligand was subsequently confirmed by molecular dynamics.

**Conclusion:**

The lead compound 16R may represent a broad-spectrum inhibitor of the 3CL^pro^ of OC43 and potentially other coronaviruses. This study provides an atomistic structure of the 3CL^pro^ of OC43 and supports further experimental validation of the inhibitory effects of 16R. These findings further confirm that the 3CL^pro^ of coronaviruses can be inhibited by broad spectrum lead compounds.

## Background

Human coronaviruses have a worldwide distribution and are frequently associated with self-limiting upper respiratory tract disease or “the common cold”. They can, however, also present with high morbidity outcomes of the lower respiratory tract including bronchiolitis, pneumonia, [1-3], asthmatic exacerbations [4] and acute exacerbations of chronic obstructive pulmonary disease (COPD) [5], where human coronavirus OC43 has been shown to be the prevalent strain in several regions [6]. Given the high burden of coronaviruses to human health, and their potential for genetic recombination to give rise to the emergence of completely novel strains, the presence of antiviral strategies is paramount. However, there is currently no commercially available molecular entity which is capable of inhibiting infection with human coronaviruses [7]. The 3CL^pro^ has been validated as an effective drug target in several studies and has even been termed “the Achilles’ heel of coronaviruses” [8] making it an ideal target for the identification of novel lead compounds.

During coronavirus replication the large open reading frame (ORF) 1a/1ab genes, located at the 5’ end of the genome, are responsible for expressing two large replicase polyproteins (pp). These are co- or post-translationally cleaved by the virally encoded 3CL^pro^ and Papain-like protease to yield 16 non-structural proteins responsible for viral replication [9]. The 3CL^pro^ is also termed the main protease as it cleaves a total of 11 cleavage sites within pp1a and pp1ab [10], in comparison to only three cleavage sites predicted for the papain-like protease [11].

The 3CL^pro^ has three distinct domains. Domains I and II are largely composed of several anti-parallel β barrels [12] and is connected to domain III by a long loop region. Domain III is composed of several globular α-helices and plays a role in protein dimerization, along with the N-terminal N-finger (residues 1–7). The active site is located in a chymotrypsin-like fold between domains I and II and contains a catalytic dyad of His41, which acts as a proton acceptor, and Cys144/5, which undergoes nucleophilic attack on the carbonyl carbon of the substrate [8,13]. The 3CL^pro^ is so named for its close structural and sequence homology to the 3 chymotrypsin protease (3C^pro^) of rhinoviruses, which contains a catalytic triad composed of His, Cys and Glu or Asp. Superimposition of the structures of 3CL^pro^ and 3C^pro^ shows that the His and Cys of both proteases is almost perfectly aligned, which may explain the similar substrate specificity and catalytic mechanism [14]. The position of the Glu/Asp in 3C^pro^ is, however, replaced by a water molecule in 3CL^pro^, which shares three hydrogen bonds with surrounding residues including the catalytic dyad member His41. It is assumed that this water molecule is responsible for stabilization of the histidine in the intermediate state during enzymatic cleavage [8].

All coronavirus 3CL^pro^ share a high sequence homology, as well as main chain architecture and substrate conservation [15,16]. The substrate binding site of the 3CL^pro^ has two deeply buried S_1_ and S_2_ subsites, with shallow S_1’_, S_3_ and S_4_ subsites with varying degrees of solvent exposure. Substrate specificity of coronavirus 3CL^pro^ is mainly determined by the P_1_, P_2_ and P_1’_ positions [16]. The P_1_ position has an absolute specificity for glutamine which stabilizes the S_1_ subsite via a hydrogen bond with the imidazole Nε2 of His162/3 and van der Waals interactions with surrounding residues of the S_1_ pocket. The P_2_ site has a preference for leucine or methionine to fill the hydrophobic S_2_ pocket. The sidechains of the S_3_ site are solvent exposed and therefore this site is expected to tolerate a wide range of functionality, but shows a preference for basic residues [17]. Sidechains and backbones of residues surrounding the S_4_ site create a highly congested pocket which favors a small, hydrophobic residue in the P_4_ position, either Ser, Thr, Val or Pro [17-19]. The S_1’_ and S_2’_ subsites also accommodate small residues in the P_1’_ and P_2’_ positions, which may include Ser, Ala or Gly [18,20]. A typical cleavage recognition site is therefore (Ser, Ala)-(Val, Thr)-Leu-Glu ↓ (Ser, Ala, Gly), which is conserved among all coronavirus 3CL^pro^ [21]. Theoretically, since these viruses share a highly homologous binding site and substrate conservation, it is reasonable to anticipate that broad spectrum inhibitor strategies might be successful. [13].

A noncovalent, furyl amide ligand, 16R, which was identified by a high throughput screen, with subsequent lead optimization based on structure-activity relationships within the S_1_, S_1’_ and S_2_ binding pockets was shown to inhibit the 3CL^pro^ of SARS-CoV at an IC50 of 1.5 ± 0.3 μM [12]. This study will detail the generation of a theoretical model of the OC43 3CL^pro^ based on a high sequence homology with the solved 3CL^pro^ structure of HKU1 and assess the potential of the inhibitor, 16R, to inhibit this complex in a broad-spectrum manner. This study will therefore provide further evidence to support the identification of broad spectrum 3CL^pro^ inhibitors.

## Results and discussion

### Homology modelling of the OC43 3CL^pro^

**Figure 1:**
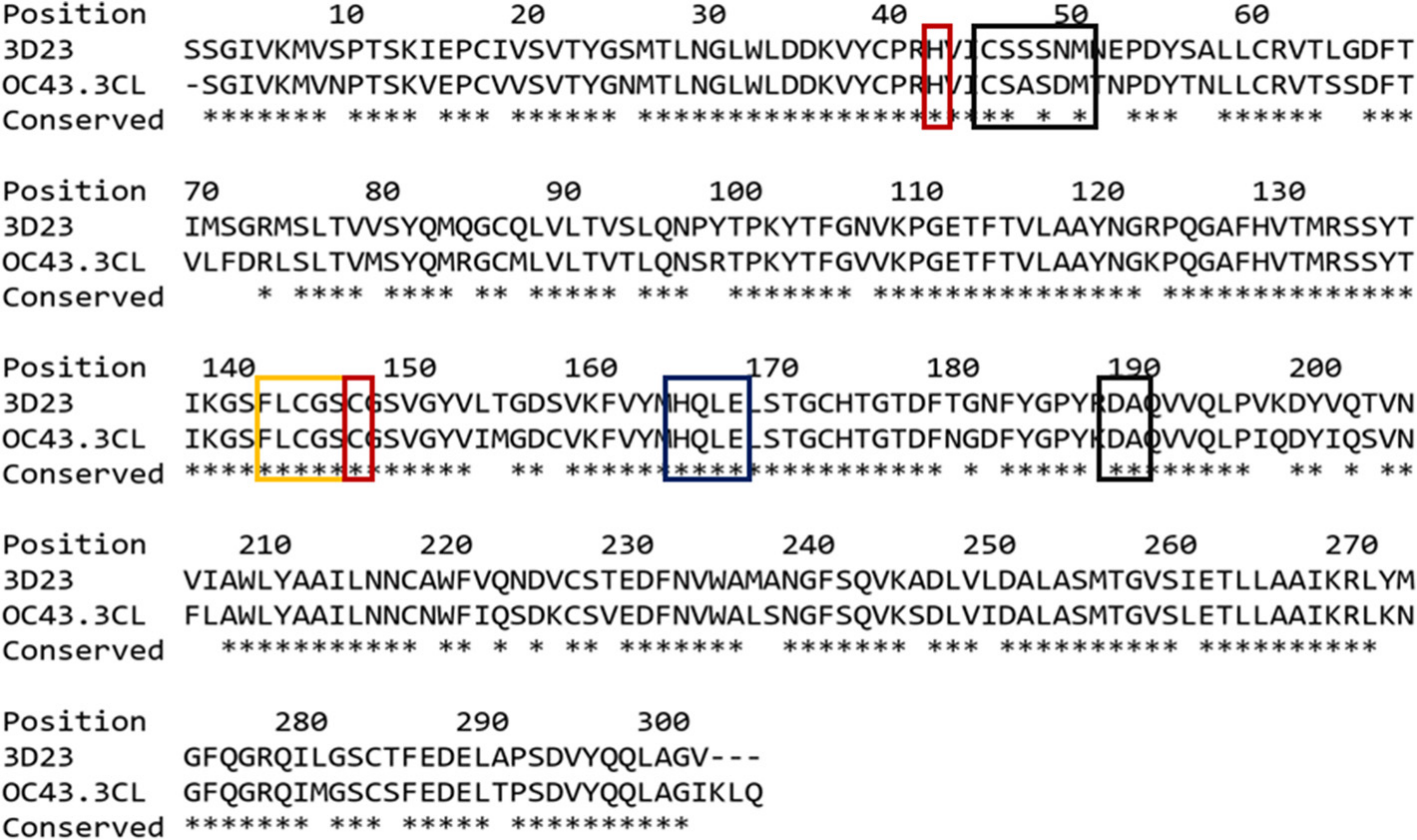
Pairwise sequence alignment of OC43 3CL^pro^ with the template structure of 3D23. Sequence alignment revealed a high identity of 82.3%. Asterisks indicate conserved residues between target and template. Conserved active site residues are highlighted in red. Important residues within the oxyanion loop (yellow), S_1_ pocket (blue) and S2 pocket (black) are also highlighted to display high degree of conservation within the active site.

The 3CL^pro^ structure of HKU1 [PDB: 3D23] displayed a high sequence identity of 82.3% to the 3CL^pro^ of OC43, with an e-value of 0.0. The high degree of identity can be partly expected as both OC43 and HKU1 are human coronaviruses from the *Betacoronavirus* genus. The exceptionally high degree of identity may even further suggest a recent common ancestor, which has yet to be identified. The active site residues are also highly conserved between both sequences indicating that 3D23 forms a highly suitable template for model generation (Figure 1).

**Figure 2:**
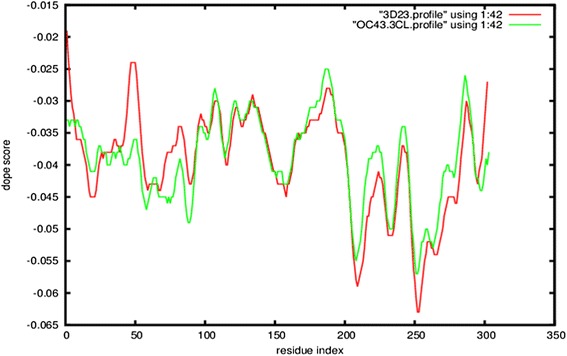
DOPE score profiles of template, 3D23, and homology model of OC43 3CL^pro^ **.** General overlay of profiles indicates the generated model is close to its native structure. The spike at residue 50 corresponds to a variable loop structure for which OC43 3CL^pro^ has assumed a more stable conformation.

**Figure 3:**
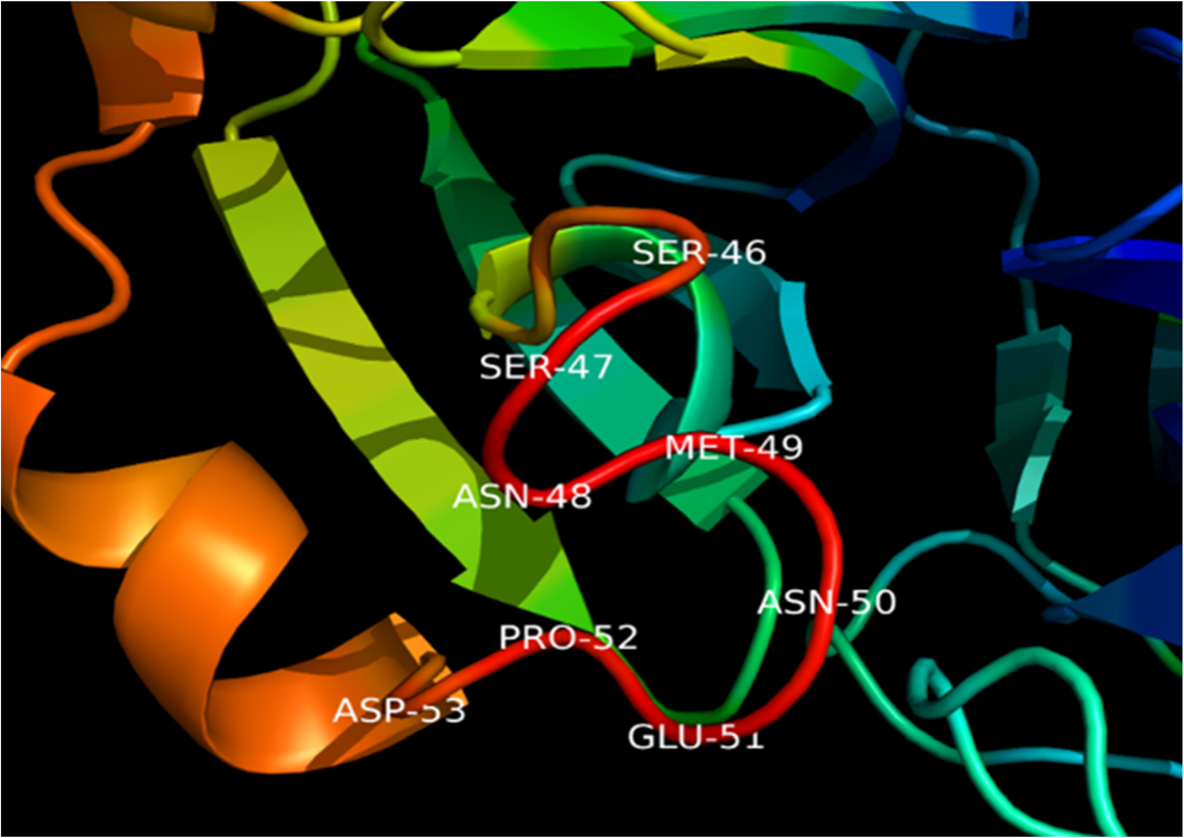
Location of highly variable loop region in the 3CL^pro^ of HKU1 (3D23). Colouring of backbone elements by B-factor indicates the presence of a highly variable loop from residues Ser46 to Asp53. This variable loop region may be responsible for the spike in DOPE score for 3D23, for which OC43 3CL^pro^ has assumed a more stable conformation.

**Figure 4:**
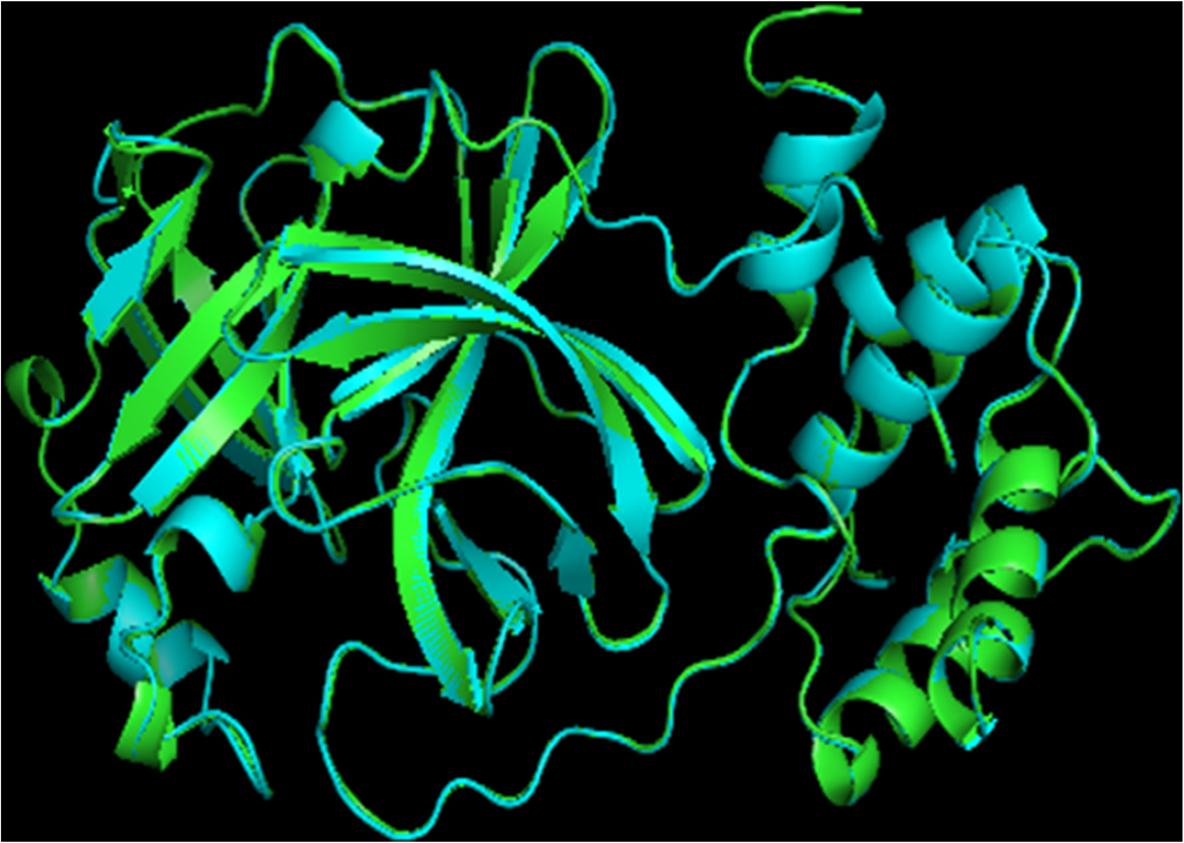
Superimposition of 3D23 and homology model of OC43 3CL^pro^. Blue ribbon represents the template structure of 3D23 with green representing the homology model of OC43 3CL^pro^. Superimposition shows a high degree of structural homology with a low RMSD of 0.327 Å.

**Figure 5:**
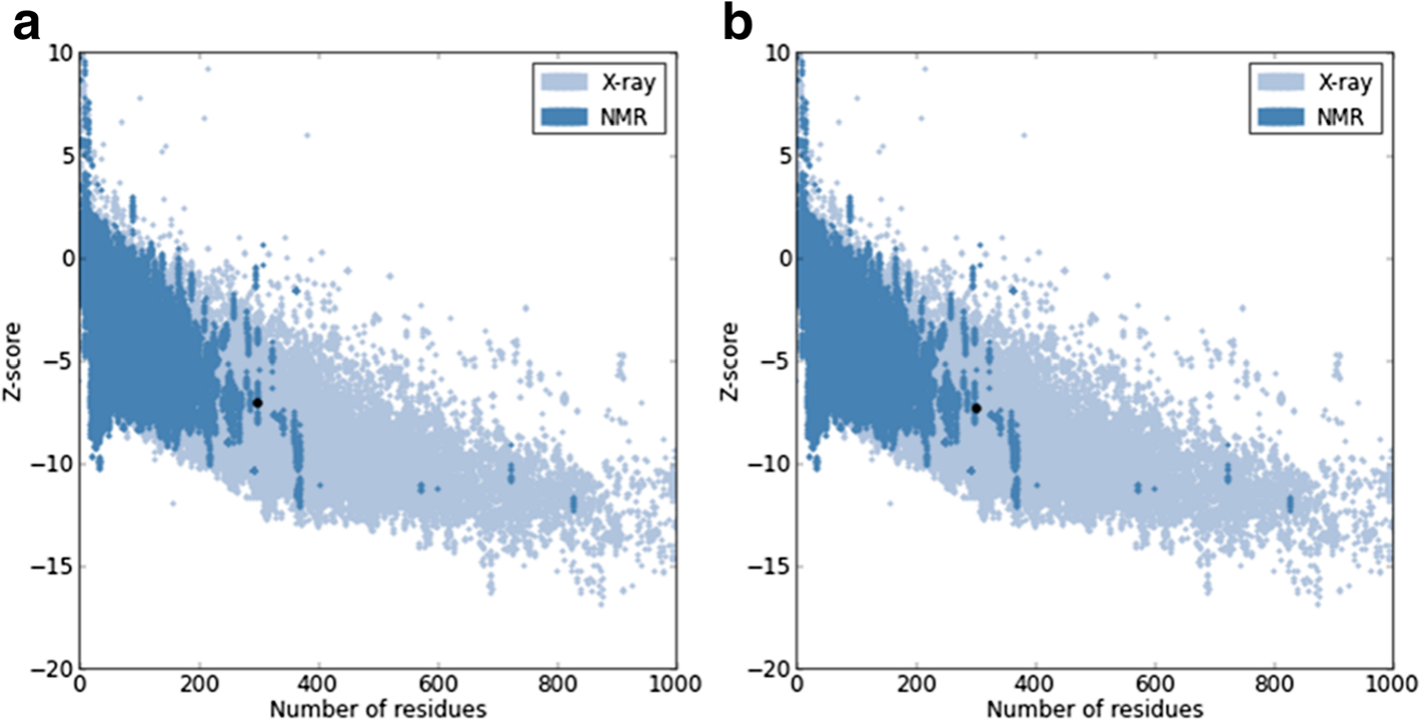
Overall quality of the model as assessed by Z-score. (**a**) Z-score for crystallographic model of 3D23. (**b**) Z-score for the homology model of OC43 3CL^pro^. A strong correlation between template and target structures indicates an accurate model.

**Figure 6:**
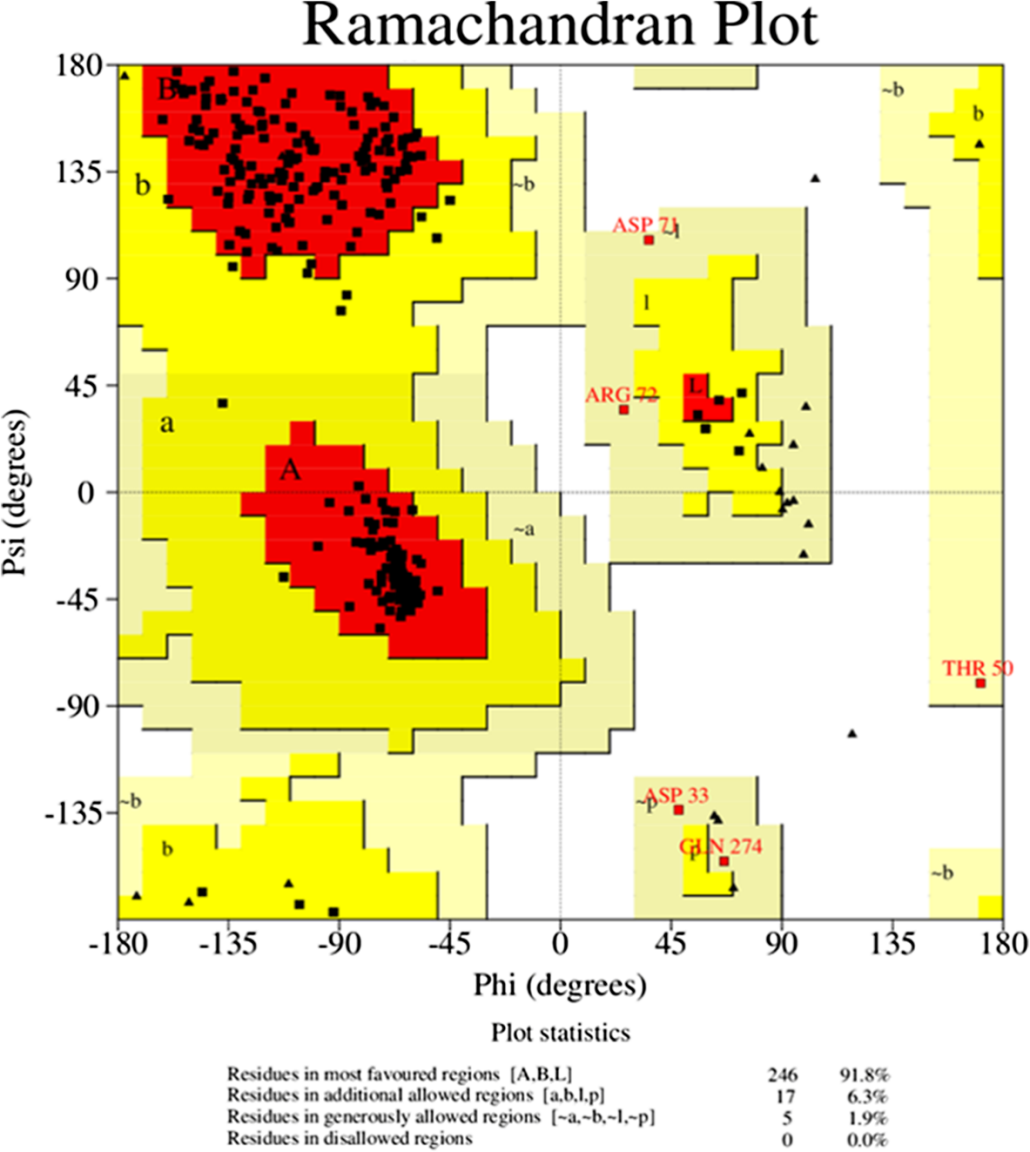
Stereochemical analysis of phi-psi dihedral angle of the OC43 3CL^pro^. Ramachandran plot generated by PROCHECK indicates that 91.8% of residues are located in the most favoured regions with none in the disallowed regions. Based on analysis of 118 solved structures, a good model is expected to have above 90% of residues in regions A, B and I [35].

Homology models were built with MODELLER (9v10) [22,23] where the lowest discrete optimized protein energy (DOPE) score corresponded to model five with a GA341 score of 1, indicating that the model quality corresponded with low resolution crystallographic structures. The DOPE score profile of target and template (Figure 2) were nearly perfectly overlaid, indicating that the model was close to its native state. A peak in DOPE score for HKU1 3CL^pro^ (3D23) was observed at approximately residue 50, where OC43 3CL^pro^ showed a moderate conservation in DOPE score. Colouring the HKU1 3CL^pro^ (3D23) structure by B-factor indicates the presence of a highly variable loop region from Ser46 to Asp53 (Figure 3). The presence of this highly variable loop structure could explain the increase in the DOPE score profile in this region and may suggest that the homology model has assumed a more stable conformation than the template. Structural alignments where the root mean square deviation (RMSD) is below 2 Å between target and template indicates that the positions of all backbone elements are correct [24,25]. Superimposition of the 3D23 template and modelled OC43 3CL^pro^ structure displayed an RMSD of 0.327 Å suggesting a highly accurate prediction of the position of all backbone elements (Figure 4). Analysis of the overall model quality of target and template by ProSA Z-score indicated that both fall within an acceptable range for crystallographic structures with a Z-score for 3D23 of −7.04 and −7.34 for the homology model of OC43 3CL^pro^ (Figure 5). Stereochemical analysis of phi-psi dihedral angles indicated that 91.8% of residues were in the most favoured regions with none in the disallowed regions (Figure 6).

For the latter purposes of this model, in characterization of the inhibitory potential of 16R, it is essential to confirm that the receptor in its active conformation. To ensure the modelled 3CL^pro^ of OC43 is in its active conformation, several features can be used to differentiate between the active and inactive states. These features include the maintenance of a loop conformation of the loop connecting domain II and III and the oxyanion loop. If these features assume an alpha helical conformation it directly or indirectly leads to the collapse of the oxyanion hole and thereby forms an inactive state [26]. An alpha helix conformation of residues 186–190 in the loop region between domain II and III causes a further collapse of the S_2_ and S_4_ subsites [19]. The orientation of the His163 sidechain in the S_1_ pocket is also vital for substrate binding [27]. This residue is largely kept in place by a stacking interaction with the benzene ring of Phe140 and imidazole ring of His163 [26]. The orientation of His172 is also stabilized by a hydrogen bond with the sidechain of Glu166. This prevents steric interactions between His172 and His163 allowing it to maintain its orientation in the S_1_ pocket. A hydrogen bond between His163 and Tyr161 has also been implicated in stabilizing the sidechain of His163 [27], however we could not observe the formation of this bond in the crystal structure of SARS-CoV 3CL^pro^ (3V3M), raising the question of its importance to maintain the orientation of His162/3.

**Figure 7:**
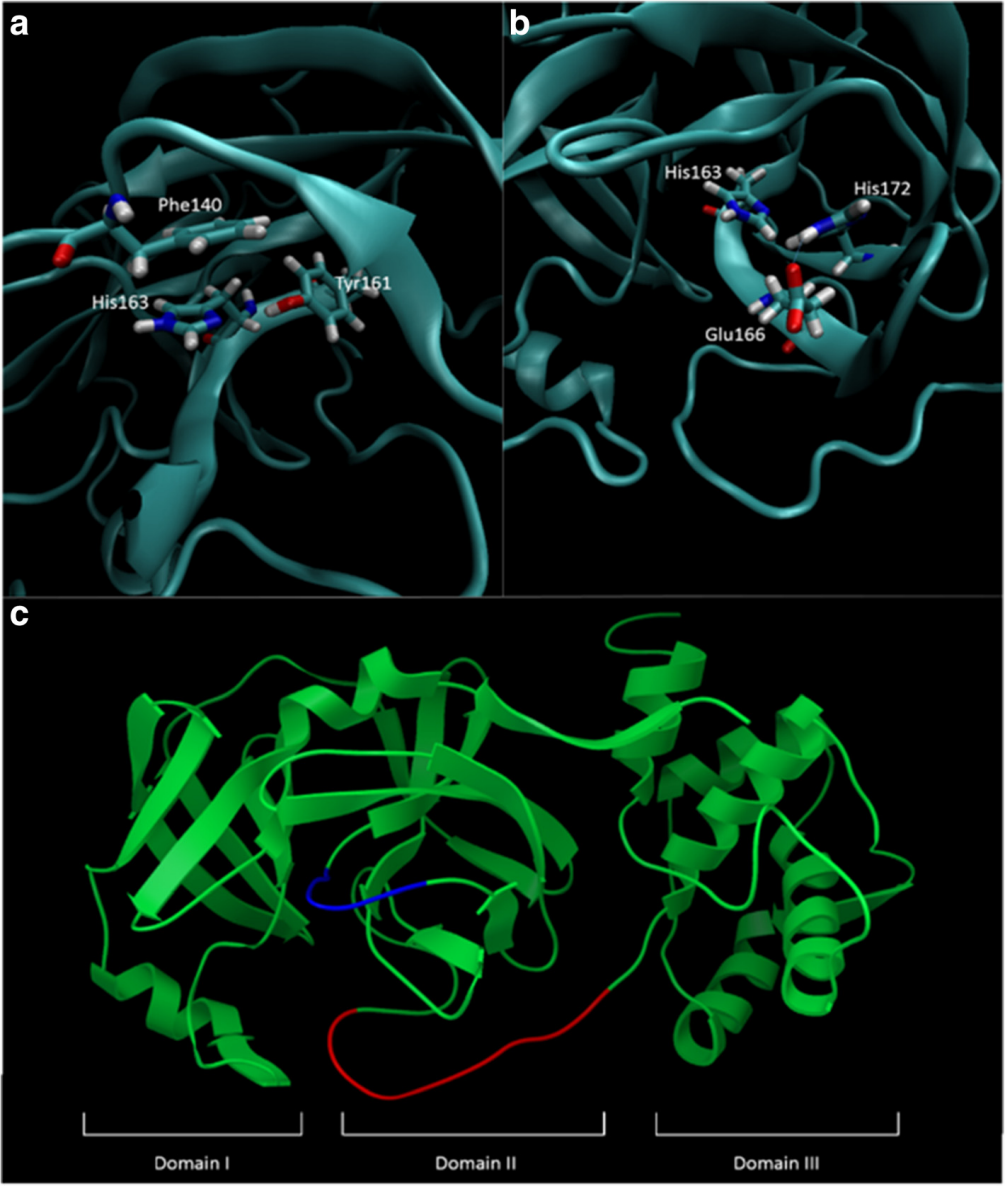
Features present in the homology model of OC43 3CL^pro^ which represent of an active state of the enzyme. (**a**) Orientation of His163 is essential for substrate binding. This orientation is maintained by stacking interactions with Phe140 and a hydrogen bond with Tyr161. The importance of this bond is questionable as it is not observed in all crystallographic models. (**b**) Steric interactions between His172 and His163 disrupt the active conformation of His163. To prevent this, His172 is stabilized by a hydrogen bond with Glu166. (**c**) Maintenance of loop structures of the oxyanion loop (blue) and the loop connecting domain II and III (red) are essential in stabilizing the oxyanion hole. The general three domain structure of all 3CL^pro^ is also depicted.

Utilising these previously mentioned parameters it is possible to ascertain if the generated homology model of OC43 3CL^pro^ is in an active conformation. With the exception of the hydrogen bond between Tyr161 and His163, which may form in a dynamic system as the Tyr161 hydroxyl is in close proximity to the imidazole nitrogen of His163 but with incorrect geometry to form a hydrogen bond, all other interactions and conformations were maintained indicating that the homology model generated using the 3CL^pro^ structure of HKU1 (3D23) as a template is representative of the active conformation of the enzyme (Figure 7). With this and previously mentioned results, indicating the generation of an appropriate homology model of the OC43 3CL^pro^, suggests the generated structure is suitable for further structure-based drug design techniques.

### Assessing the binding conformation and free energy of binding of 16R

**Figure 8:**
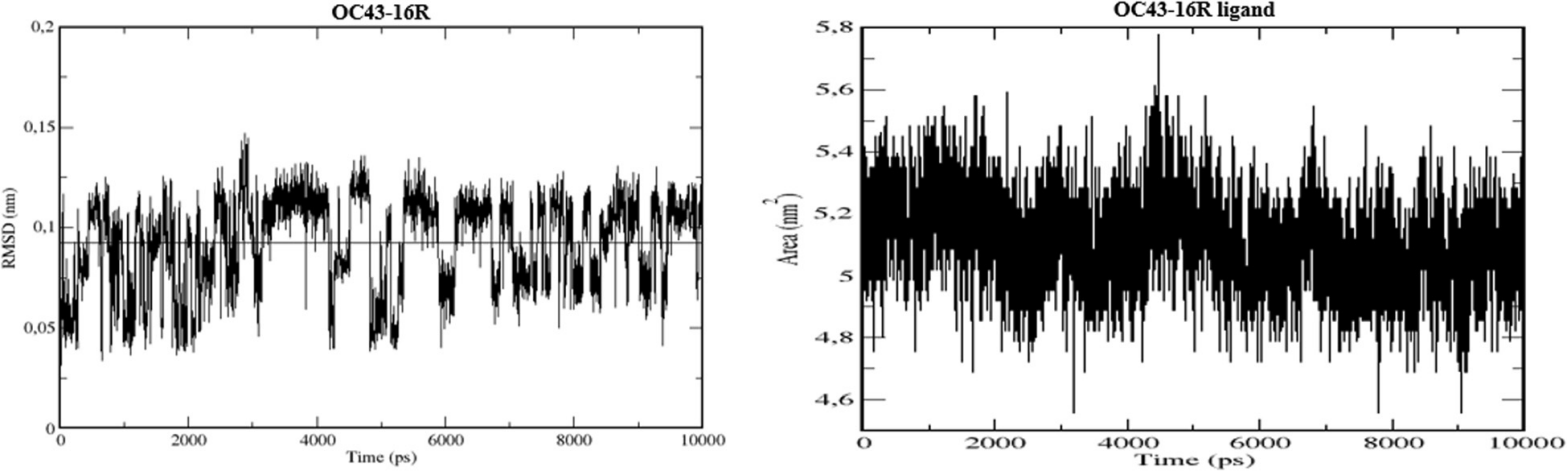
Binding conformation of 16R within the active pocket of the 3CL^pro^. (a) Pose of 16R within the active site of the SARS-CoV 3CL^pro^. Hydrogen bonds (yellow dashes) are formed with Gly143, His163 and Glu166. (b) 16R assumes a similar pose when bound to the OC43 3CLpro. A notable difference is the loss a hydrogen bond with Glu166. Hydrogen bonds with Gly143 and His163 are however maintained.

Analysis of the crystallographic structure of the SARS-CoV 3CL^pro^, with the bound inhibitor 16R, illustrates a pyridine occupying the S_1_ pocket and forming a hydrogen bond with the imidazole Nε2 of His163. A further two hydrogen bonds are formed between ligand carbonyls and Gly143 and Glu166 with three methyl groups inserting into the deep hydrophobic S_2_ pocket [12]. The conformation of 16R is almost completely conserved when bound to the 3CL^pro^ of OC43. Notable exceptions include the shifting of the hydrogen bond formed with Gly143 to a furan ring oxygen as opposed to the carbonyl oxygen seen in SARS-CoV 3CL^pro^. The hydrogen bond formed with Glu166 is also absent in OC43 3CL^pro^, however the distance between the ligand carbonyl and backbone hydrogen is 2.75 Å and therefore this may be capable of mediating hydrogen bond formation in a dynamic system (Figure 8). Free energy of binding between 16R and SARS-CoV and OC43 3CL^pro^, as assessed by MM-GBSA, is -85 kcal/mol for both receptors. These results suggest that the inhibitor 16R may be capable of inhibiting both complexes.

### Molecular dynamic simulation of OC43 and 16R

To further confirm the findings described in the previous section, and to assess the potential of Glu166 to form a hydrogen bond in a dynamic system, molecular dynamic simulations were used. Molecular dynamics of the SARS-CoV 3CL^pro^-16R complex are in strong agreement with the crystal data of 3V3M [12]. The simulation predicts an average of 3.17 hydrogen bonds formed over the 10 ns trajectory, with these bonds predominantly forming with Glu166, His163 and Gly143. The bond formed at Gly143 does however display the potential to form a bifurcated interaction with Asn142. As predicted by the previous results, detailed in the section describing the binding conformation, the 3CL^pro^-16R complex of OC43 displayed a high occupancy of 100% and 85% at both His163 and Gly143 positions respectively. These results however suggested that the bond formed with Gly143 was mediated via the furan ring oxygen, where molecular dynamic simulations indicated that this bond was formed between the carbonyl oxygen and Gly143, as seen with the crystal structure of SARS-CoV. Molecular dynamics also confirmed that the hydrogen bond at the Glu166 does not stably form in a dynamic environment and only displays the potential to form at 0-2 ns and 4-5 ns, with an overall occupancy of 21.62%.

**Figure 9:**
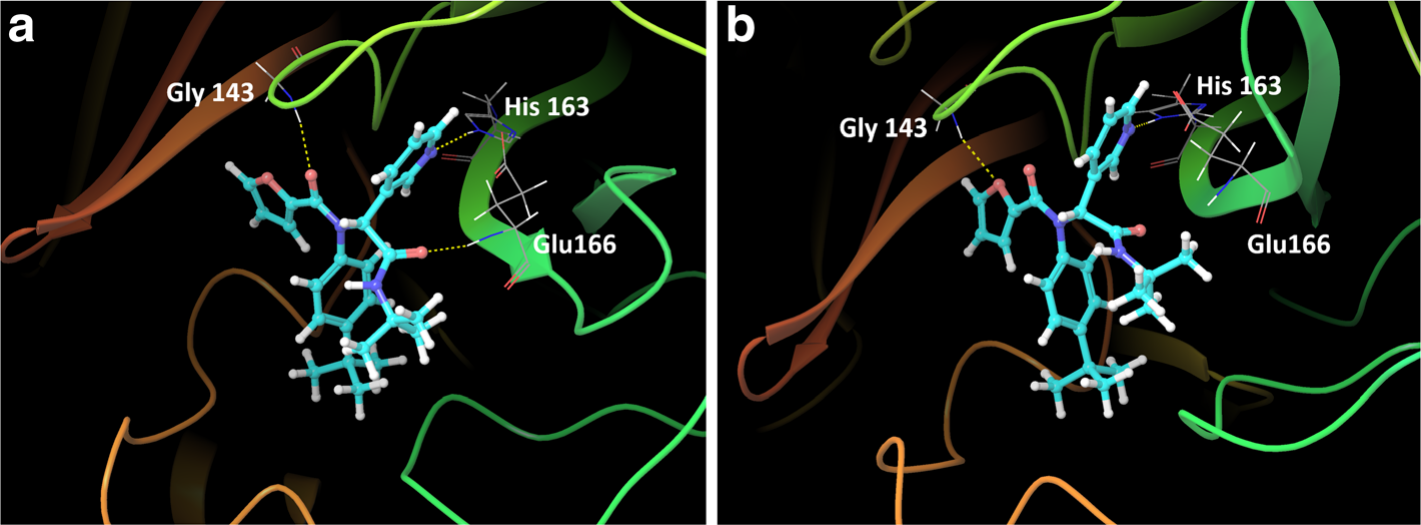
RMSD and solvent accessible surface area of 16R when bound to the OC43 3CL^pro^. Analysis of ligand RMSD and solvent accessible surface area depicts a ligand which is stably bound to its receptor.

The stability of the bound ligand within the active pocket was also assessed via RMSD, radius of gyration and changes in solvent accessible surface area. A RMS deviation below 2 Å is an indication of a ligand which is stably bound to its receptor [28], where an increase in radius of gyration from the starting reference would suggest ligand instability. An increase in solvent accessible surface area of the ligand may indicate the ligand is dissociating into surrounding solvent [29]. Ligand RMSD within the active site was approximately 0.75 Å and radius of gyration and solvent accessible surface area remained within a stable range of 3.9 Å to 4.0 Å and 4.8 nm^2^ to 5.4 nm^2^ respectively (Figure 9). These parameters suggest that the ligand is highly stable within the active site.

## Conclusion

The three-dimensional structure of the OC43 3CL^pro^ was successfully solved by homology modelling with MODELLER. Analysis of various side chains and loop conformations within and surrounding the substrate-binding site is indicative of an active conformation of the enzyme. The solved structure has been made publicly available in the Protein Model Database (PMDB; https://bioinformatics.cineca.it/PMDB/) [PMDB ID: PM0079872]. Further analysis of a previously identified lead compound, 16R, suggests that it makes extensive and stable interactions with the 3CL^pro^ binding site of OC43. Additional analysis of the free energy of binding by MM-GBSA suggests that the ligand binds with the same affinity to the 3CL^pro^ of OC43 as it does to SARS-CoV, a receptor it is experimentally proven to inhibit. These results are strongly suggestive that the lead compound, 16R, not only displays the potential to inhibit the 3CL^pro^ of SARS-CoV but also that of the highly homologous OC43. In additional studies (data not shown) 16R was shown to remain stably bound to the 3CL^pro^ of 229E and NL63 in molecular dynamic simulations and may therefore even display the potential to inhibit the 3CL^pro^ of additional coronaviruses. 16R is therefore an excellent lead compound in the pursuit of true broad spectrum inhibitors of all coronaviruses.

## Methods

### Homology modelling of the OC43 3CL^pro^

The amino acid sequence of the OC43 3CL^pro^ was obtained from the NCBI database [GenBank: AEN19363]. Fold assignment with the *profile.build* module of MODELLER (9v10) identified the most suitable template structure from PDB with an 82.3% sequence identity and e-value of 0. Template-target alignment was conducted with the *align.2d* module, which also incorporates structural information from the template. Models of the OC43 3CL^pro^ were built by satisfaction of homology derived spatial restraints on distances and dihedral angles [23], stereochemical restraints obtained from the CHARMM22 force field [30] and statistical preferences on dihedral angles and non-bonded distances obtained from known protein structures [22,31]. Models were built by the automodel class by minimizing the violations on all restraints and united-atom PDB models were generated.

DOPE scores are assigned by considering the positions of all non-hydrogen atoms, where the lowest DOPE score corresponds to the model that is predicted to be most accurate [31]. The *evaluate_model.py* script was further utilised to calculate per residue DOPE scores, which were superimposed over the template structure DOPE scores. A general conservation in the pattern of respective DOPE scores was used as an indication of an accurate model. A low RMSD, as assessed by PyMOL [32], indicated structural homology between solved structure and template. The GA341 score was used to analyse fold-assignment. Z-scores, obtained from ProSA-web [33,34] were used to analyse overall model quality and to assure template and query models were in a range acceptable for structures of their respective size. A Z-score outside a range characteristic for native proteins will suggest the production of an erroneous model [33]. Stereochemical analysis of dihedral angles was then further conducted using PROCHECK [35,36] on models corresponding to the lowest DOPE score with GA341 scores amounting to 1.0. Stereochemical analysis by PROCHECK assessed residue geometry, where more than 90% of residues should be located in the “most favoured region” to indicate a stereochemically favourable structure.

Once an appropriate model accuracy was achieved based on these previous observations, the active site of the enzyme was analysed to ensure it was in its active state. Several features are able to differentiate an active from inactive state of the 3CL^pro^. These include the conformation of the oxyanion loop and loop connecting domain II and III [19,26] and the sidechain orientation of residues, including His163, Phe140, His172, Tyr161 and Glu166, within the S_1_ pocket [26,27].

### Assessing the binding conformation and free energy of binding of 16R

Protein structures of 3V3M (3CL^pro^ of SARS-CoV) and the homology modelling derived structure of OC43 3CL^pro^ were prepared with the Schrӧdinger Protein Preparation Wizard. Histidine protonation was assigned to the epsilon nitrogen in accordance with crystal data. A restrained minimization was then performed using the OPLS2005 force field [37,38], during which heavy atoms were restrained to remain within 0.3 Å of the original structure. To assess the conformation of the known inhibitor, 16R, when binding to the OC43 3CL^pro^, the experimental structure of 3V3M and OC43 3CL^pro^ were aligned in Maestro, the interface for all Schrӧdinger’s software. The inhibitor, 16R, was subsequently superimposed over the OC43 3CL^pro^ active site using coordinates present in the 3V3M crystal structure. Side chains of residues in the active pocket assuming orientations that resulted in a steric clash with the ligand were refined and the ligand was minimized with Prime [39,40] to better fit the pocket. Prime utilizes both empirically and theoretically derived constraints to predict and replicate the dynamic motion of protein sidechains, allowing for conformational changes that arise through the “induced-fit” phenomenon, which is often neglected in molecular docking screens.

The Prime/MM-GBSA method was used to calculate the binding free energy of 16R within the respective receptor. The free energy of binding is the calculated difference between the minimized receptor-inhibitor complex and the sum of the energies of the minimized unbound inhibitor and receptor. Ligand poses were minimized using the local optimization feature in Prime, where energies of the complex were calculated with the OPLS-2005 force field and GBSA continuum solvent model.

### Molecular dynamic simulation of the 3CL^pro^-16R complex

The CHARMM27 all atom force field was assigned to receptor structures using the three point TIP3P water model. All input hydrogens in the coordinate file were ignored and were assigned according to the force field. Histidine protonation states were assigned to the epsilon nitrogen in the neutral form in accordance with crystallographic data. The ligand, in the previously predicted pose, was prepared by SwisParam [41]. The protein-ligand complex was then placed in the centre of a solvated, cubic box, 1 nm from the box edge. The system was solvated with the spc216 water model and sodium counter ions were added to neutralize the system. The system was energy minimized by a brief dynamics simulation using Steepest Descent Minimization until steepest descent converged to Fmax < 1000. Long range electrostatic interactions were calculated with the Particle Mesh Ewald (PME) method [42] with a cutoff of 1 nm and periodic boundary conditions were applied. Both NVT and NPT equilibrations were run for 50 000 steps or 100 ps using a 2 fs time step and a leap-frog integrator. All bonds were constrained by the lincs algorithm. PME was again used for long range electrostatics with a 0.16 fourier spacing. Short range electrostatic and van der Waals cutoffs were 1.0 nm and 1.4 nm respectively. Coordinates, velocities and energies were saved every 0.2 ps. During NVT equilibration temperature coupling was achieved by V-rescale algorithm with a target temperature of 300 K. Pressure coupling during NPT equilibration was achieved via the Parrinello-Rahman algorithm and is incorporated in the NPT equilibration once temperature is stable to ensure the proper density is reached (approximately 1000 kg/m^3^). Following NVT and NPT system equilibration an unrestrained, 10 ns simulation (5 000 000 steps) was run with the leap frog integrator, saving coordinates, velocities and energies every 2 ps. All other parameters were maintained from the NVT and NPT equilibration including both temperature and pressure coupling.
